# Microvascular decompression in trigeminal neuralgia with the offending artery transfixing the nerve: a case report

**DOI:** 10.1186/s12883-022-02765-4

**Published:** 2022-07-04

**Authors:** Xinyu Zhang, Yang Li, Mi Zhou, Zhenqing Wei

**Affiliations:** 1 Department of Neurosurgery, The First Affiliated Hospital of Dalian Medical University, Dalian, Liaoning China; 2 Department of Neuroelectrophysiology, The First Affiliated Hospital of Dalian Medical University, Dalian, Liaoning China

**Keywords:** MVD, TN, Treatment, Transfixing, Case report

## Abstract

**Background:**

An anterior inferior cerebellar artery (AICA) that crosses the right trigeminal nerve is an uncommon arterial anatomic variation. In this anatomical position, it is difficult to separate or move the offending blood vessels and nerves. We report an uncommon case of trigeminal neuralgia (TN) caused by compression of the trigeminal ganglion by a branch of the AICA.

**Case presentation:**

A 34-year-old man with 5 years history who complained of pain on the right side of the face (area V1). The symptoms gradually worsened, and the pain episodes became intense and frequent. Magnetic resonance imaging (MRI) of the cerebrum showed a small blood vessel passing through the right trigeminal nerve. Microvascular decompression (MVD) was performed,because medication was ineffective. Intraoperative exploration confirmed that the vessel which was a branch of the AICA passing through the right trigeminal nerve. As while the artery was temporarily clipped, electrophysiological monitoring showed a decrease in the amplitude of nerve activity. As the artery was considered too important to be sacrificed, the space between the nerves was enlarged mildly, the artery was liberated, the Teflon implant was shredded and placed between the artery branches and nerve to make the blood vessels as perpendicular as possible to the nerve. The patient had no neurological dysfunction and no pain after 8 months of follow-up.

**Conclusion:**

MVD is an effective treatment for artery-induced trigeminal nerve compression, but we report a novel procedure that avoids the complication of facial numbness caused by cutting the offending vessels and incision of the trigeminal nerve.

## Background

Vascular compression of the fifth cranial nerve in the cerebellopontine angle commonly as the cause of TN have drawn the attention of neurosurgeons. However, sometimes TN is also be induced by idiopathy or lesions including cysts [1], tumors [2], arteriovenous malformation (AVM) [3] and multiple sclerosis [4].

Based on the consensus of vascular compression of the fifth cranial nerve can caused TN.MVD has been generally considered the standard microsurgical treatment for TN. The superior cerebellar artery (SCA) is the most common causative vessel, and other vessels involved either alone or in conjunction with other vessels were the AICA, the vertebral artery, and the petrosal vein. A transfixing artery has rarely been reported in the literatureas the offending vessel in TN [5]. The tasks of separating the responsible vessels from the nerves and relieving compression by the vessels become extremely difficult when an interneural vessel is the offending vessel because sufficient MVD may force the operator to perform accessory injury in the anatomic condition.

The authors report a patient with TN caused by a penetrating artery; the patient was successfully treated by microvascular decompression. In addition, the authors reviewed the relevant literature.

## Case presentation

**Fig. 1:**
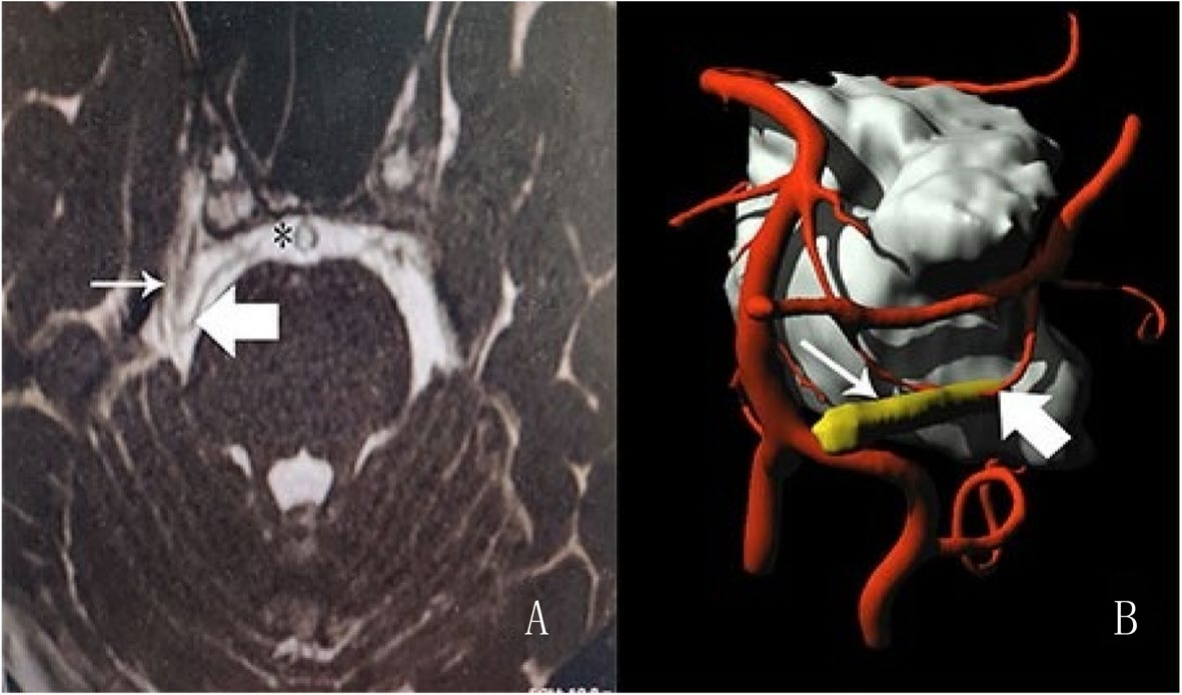
**A** The offending artery (solid arrow) and the nerve (arrow) are depicted as low-signal-intensity structures, and the cerebrospinal fluid (*) has high signal intensity. **B** Virtual reality (VR) image showing the offending intraneural artery (solid arrow) between the branches of the sensory roots of the trigeminal nerve (arrow)

A 34-year-old male had a 5-year history of facial pain in the right V1 regions. In 2016, oral carbamazepine uptake resulted in pain relief. In March 2021, the pain episodes became intense and frequent, increase the dose of carbamazepine was ineffective in relieving the pain. Notably, the patient did not have classic “trigger point pain”. Each time, the patient had a pain episode lasting 5 to 8 min, which was significantly longer than the duration of classic symptoms. In September 2021, the patient was admitted to our institute for MVD. The patient underwent high-resolution three-dimensional fast imaging employing steady-state acquisition magnetic resonance imaging (3D FIESTA MRI). The 3D FIESTA sequence can clearly visualization the neurovascular anatomy of TN patients. 3D FIESTA imaging which has high contrast and spatial resolution could depicted structures surrounded by cerebrospinal fluid (CSF). On images, There are depicted as different signal-intensity structures between blood vessels and the nerve and the CSF.MRI of the trigeminal nerves revealed a small vessel penetrating the right trigeminal nerve. Multimodal MRIs were then coregistered with open- source software 3D Slicer, and 3D image reconstruction was performed to generate virtual reality (VR) images for detecting possible penetrating arteries (Fig. 1).

**Fig. 2:**
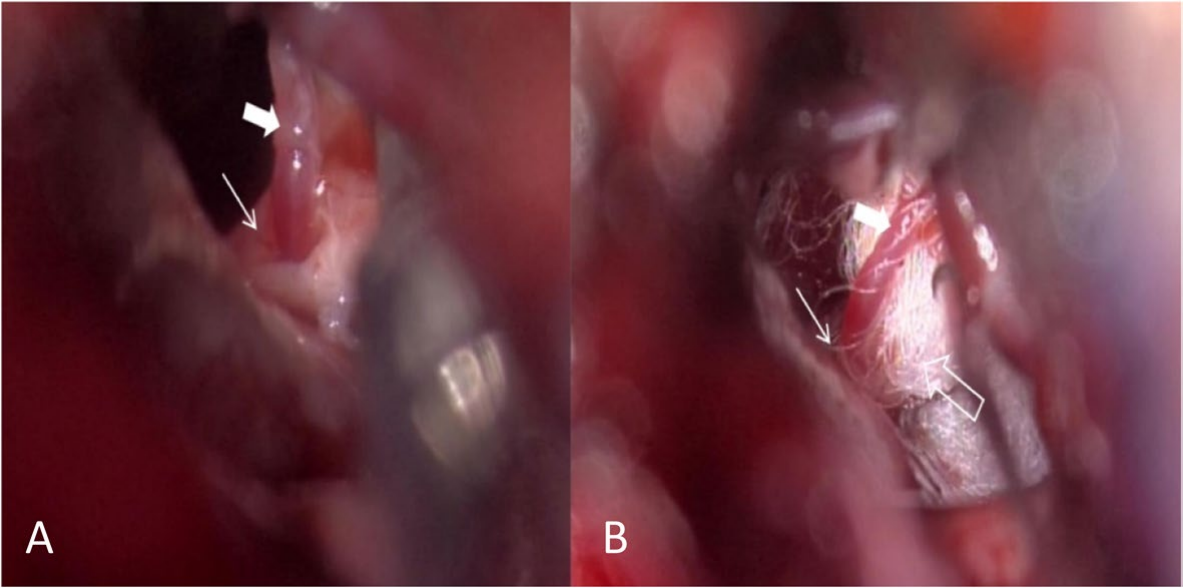
**A** Intraoperative photograph showing the offending intraneural artery (solid arrow) between the branches of the sensory roots of the trigeminal nerve (arrow). **B** Ashredded Teflon implant (arrow outline) was placed between the artery (thick arrow) and nerve branches (thin arrow) to position the artery as perpendicular as possible to the nerve

A regular retrosigmoid suboccipital craniotomy was carried out to access the cerebellopontine angle, and we confirmed that the blood vessel transfixing the right trigeminal nerve was a branch of the AICA. Furthermore, the whole course of the trigeminal nerve was carefully examined, and no other offending vessels were founded (Fig. 2A).

**Fig. 3:**
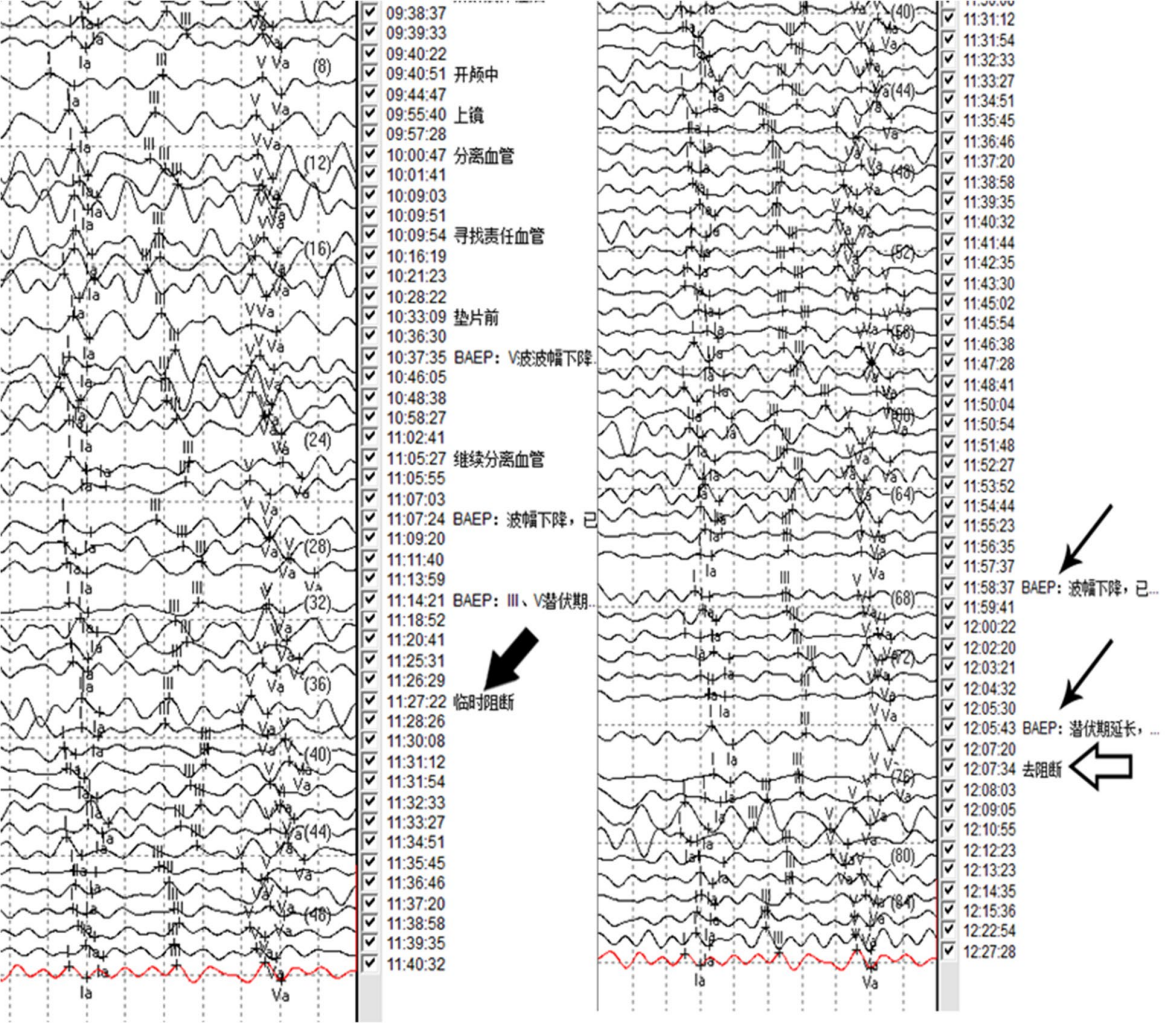
**A** The time that the offending artery was clipped was 11:27(thick arrow). **B** The electrophysiological monitoring showed a decrease in amplitude and gave two warnings to the operator (thin arrow). The clip was removed from the offending artery immediately, and the amplitude recovered in 7 min (arrow outline)

We attempted to cut the offending artery and separate it from the nerve. After 30 min of temporary occlusion of the offending artery, electrophysiological monitoring showedthat the latency of the V wave exceeded 1 millisecond, and each waveform decreased by more than 50%, indicating that the hearing pathway was damaged and that the patient was at risk of hearing loss (Fig. 3B). The clip was immediately removed from the offending artery, and thelatency and amplitude ofthe V wave recovered in 7 min (Fig. 3B). The artery was considered too important to sacrifice.

The trigeminal nerve was inspected carefully, the space between the nerves was mildly enlarged, and the artery was liberated. A shredded Teflon implant was placed between the artery and nerve branches to make the blood vessels as perpendicular as possible to the nerve. Importantly,the shredded Teflon implant was placed in the crossing space between the offending artery and nerve rather than at the passing point (Fig. 2B). The pain was relieved postoperatively, and the patient did not have any pain or neurological dysfunction at an 8-month follow-up visit.

## Discussion

MVD was first used by Gardner and Sava as a surgical treatment for TN, and it was later popularized by Peter Jannetta [6]. It has become the gold standard surgical treatment for TN and has provided consistent and excellent long-term results.

In rare cases, arteries or veins may pass through the trigeminal nerve. The passage of blood vessels through the trigeminal nerve is an uncommon cause of TN. Because of this positional relationship, it is difficult to separate or move the offending vessel and nerve. This type of compression is difficult to treat properly. Patrick [7] reported a case in which the anterior inferior cerebellar artery penetrated the middle part of the fifth cranial nerve. To prevent compression of the nerve,they separated the trigeminal nerve carefully to allow a sizeable open artery. Six months after surgery, the patient relapsed and received radiofrequency ablation.

Tashiro et al.reported the first three cases of arterial penetration into the trigeminal nerve [5]. Two of the patients had the blood vessels and nerves partially severed, producing hemianesthesia as a complication. The operator widenedthe gap between blood vessels and nerves and moved the blood vessel to the distal end. The patients were cured without complications.

Helbig divides TN into two categories, type I: The blood vessel crosses the combined sensory and motor trigeminal nerve branches between motion and sensation; type II: The blood vessel crosses the sensory branches of the trigeminal nerve [8]. Helbig et al. reported TN accompanied by veins passing through the nerve in 3 patients. After electrocoagulation, the patient achieved good pain relief for the more delicate nerve–vein crossings, with no facial numbness. For larger penetrating veins, the vessel and the nerve were separated, and ashredded Teflon implant was inserted at the intersection between the vein and the nerve. Although facial pain was relieved, the patient developed facial numbness neuropathy and a dense hypoesthesia of corneal after surgery [8].

Up to now, there is still no standard therapeutic schedule for this situation. JunyaJito and Kazuhiko Nozaki used the slinging method to shift blood vessels and nerves [9]. Slinging the artery appeared to be the best method, producing excellent follow-up results without causing collateral damage or dislodging the vessel. However, due to the narrow operating space, it is difficult to achieve the sling operation. Otherwise, repeated separation of the vessel and nerve can lead to facial numbness.

According to the existing literature, veins with a small diameter can simply be cut off. However, coagulating or cutting off a vein with a sufficiently large diameter may lead to facial numbness and corneal sensation. If the penetrating vessel is an artery, it is difficult to move the vessel for decompression. At present, the primary method is to allow free movement of the artery. The nerve root is cut to shift the artery into the pad. Alternatively, the blood vessels are moved to the distal end of the intracranial segment of the nerve root by slinging to the bone.

We communicated with the patient before the operation. The young patient indicated that he would not accept facial numbness. We observed that the artery was very small, and we attempted to sever it. The operator clipped the artery, and the amplitude of nerve activity decreased 20 min later. Immediately after the clip was removed, the amplitude returned to normal. When blocking the aneurysm, we found it impossible to cut the artery. Because cutting the nerve root is bound to cause facial numbness, we abandoned the method of cutting part of the trigeminal nerve. When inserting shredded Teflon, we were cautious of the risk of facial sensory impairment. Finally, we opened a slight gap around the vessel and used shredded Teflon to pad the space between the vessel and nerve so that these structures could maintain as little contact as possible. Postoperatively, thepatient’s pain was relieved, and no facial numbness remained. There was no recurrence after 8 months of follow-up.

## Conclusions

We describe an uncommon case of TN caused by an artery penetrating the trigeminal nerve. However, the young male patient made clear to us that facial numbness was unacceptable. To satisfy the patient’s request, we merely separated the nerve and artery and used shredded Teflon placed between the nerve and the culprit vessel to position the artery as perpendicular as possible to the nerve, which decreases the contact area between the artery and the nerve.

The main advantage of this procedure is that it does not entail cutting the offending vessels and incising the trigeminal nerve in the longitudinal or transverse direction, which can lead to facial numbness. This procedure may be the optimal choice for young patients who cannot accept facial numbness.

## Data Availability

All data related to this case report are stored at The First Affiliated Hospital of Dalian Medical University (Dalian,China) and are available from the corresponding author upon reasonable request.

## Abbreviations

AICA: Anterior inferior cerebellar artery
3D FIESTA MRI: Three-dimensional fast imaging employing steady-stateacquisition magnetic resonance imaging
CSF: Cerebrospinal fluid
VR: Virtual reality
MVD: Microvasculardecompression
TN: Trigeminal neuralgia
AVM: Arteriovenous malformation
SCA: Superior cerebellar artery
